# Supplementary material for: Structural basis for a nucleoporin exportin complex between RanBP2, SUMO1-RanGAP1, the E2 Ubc9, Crm1 and the Ran GTPase
Source: bioRxiv. 2024 Dec 26:2024.10.04.616749. Preprint. [Version 2] doi: 10.1101/2024.10.04.616749 (PMC11703149; doi:10.1101/2024.10.04.616749)
Supplement: 1 [file NIHPP2024.10.04.616749V2-supplement-1.pdf]

bioRxiv preprint doi: https://doi.org/10.1101/2024.10.04.616749; this version posted December 26, 2024. The copyright holder for this preprint (which was not certified by peer review) is the author/funder, who has granted bioRxiv a license to display the preprint in perpetuity. It is made available under aCC-BY 4.0 International license.

Supplementary Table 1. EM data and refinement

	RanBP2/Ubc9/SUMO1-RanGAP1/Ran(GTP)/Crm1/Ran(GTP) complex – K2
**Data collection**	Dataset 1 - 4
Magnification	22,500x
Voltage (kV)	300
Electron exposure (e⁻/Å²)	85.2
Defocus range (μm)	-1.0 to -3.0
Super-resolution pixel size (Å)	0.544
Fourier cropped pixel size (Å)	1.088
Initial particle projections (#)	540,834
Final particle projections (#)	
Overall map	
(All components)	534,708
Focused Refinement (Foc. Ref.)	
Crm1/Ran(GTP)	534,708
Overall map	
RanBP2-RBD4/Ran(GTP)/RanGAP1-GAP	287,278
Foc. Ref.	
RanBP2-RBD4/Ran(GTP)	287,278
RanGAP1-GAP	287,278
Overall map	
RanBP2/Ubc9/SUMO1-RanGAP1/Ran(GTP)	61,110
Foc. Ref.	
RanBP2/Ubc9/SUMO1-RanGAP1	61,110
Crm1/Ran(GTP)/Ubc9/SUMO1-RanGAP1	61,110
Symmetry imposed	C1
Map resolution (Å)	
FSC threshold = 0.143	
Overall	
(All components)	3.18
Focused Refinement (Foc. Ref.)	
Crm1/Ran(GTP)	2.89
Overall	
RanBP2-RBD4/Ran(GTP)/RanGAP1-GAP	3.52
Foc. Ref.	
RanBP2-RBD4/Ran(GTP)	3.52
RanGAP1-GAP	3.37
Overall	
RanBP2/Ubc9/SUMO1-RanGAP1/Ran(GTP)	3.40
Foc. Ref.	
RanBP2/Ubc9/SUMO1-RanGAP1	3.29
Crm1/Ran(GTP)/Ubc9/SUMO1-RanGAP1	3.10
Map resolution range (Å)	**Box (contoured)**
Overall	
(All components)	2.44-10.70 (2.59-7.39; 0.055 sig)
Focused Refinement (Foc. Ref.)	
Crm1/Ran(GTP)	2.32-9.80 (2.42-5.59; 0.050 sig)
Overall	
RanBP2-RBD4/Ran(GTP)/RanGAP1-GAP	2.57-11.86 (2.74-6.04; 0.045 sig)
Foc. Ref.	
RanBP2-RBD4/Ran(GTP)	2.67-11.33 (2.86-6.20; 0.045 sig)
RanGAP1-GAP/Ran(GTP)	2.63-11.98 (2.63-4.02; 0.045 sig)
Overall	
RanBP2/Ubc9/SUMO1-RanGAP1/Ran(GTP)	2.56-11.82 (2.70-8.58; 0.035 sig)
Foc. Ref.	
RanBP2/Ubc9/SUMO1-RanGAP1	2.96-11.11 (2.95-6.05; 0.025 sig)
Crm1/Ran(GTP)/Ubc9/SUMO1-RanGAP1	2.50-10.48 (2.69-6.52; 0.045 sig)
**Refinement**	
Initial models used (PDB code)	3UIN, 3GJX, 4L6E, 1K5D
Model resolution (Å) FSC threshold = 0.5	2.97
Map sharpening B factor (Å²)	
Overall map	
(All components)	-82.11
Focused Refinement (Foc. Ref.)	
Crm1/Ran(GTP)	-63.36
Overall map	
RanBP2-RBD4/Ran(GTP)/RanGAP1-GAP	-135.37
Foc. Ref.	
RanBP2-RBD4/Ran(GTP)	-142.82
RanGAP1-GAP	-154.44
Overall map	
RanBP2/Ubc9/SUMO1-RanGAP1/Ran(GTP)	-55.18
Foc. Ref.	
RanBP2/Ubc9/SUMO1-RanGAP1	-86.25
Crm1/Ran(GTP)/Ubc9/SUMO1-RanGAP1	-53.40
**Model composition**	
Non-hydrogen atoms	19,287
Protein residues	2,414
Nucleic acid residues	2
Ligand (Mg^{2+})	2
**Mean *B* factors**	
Protein	62.9
Nucleic acid	40.8
Ligand (Mg^{2+})	43.4
**RMS deviations**	
Bond lengths (Å)	0.003
Bond angles (º)	0.447
**Validation**	
Molprobity score	1.43
Clashscore	4.14
Rotamer Outliers (%)	1.54
C-beta deviations (%)	0
**Ramachandran plot**	
% favored	92.11
% allowed	7.89
% outliers	0

bioRxiv preprint doi: https://doi.org/10.1101/2024.10.04.616749; this version posted December 26, 2024. The copyright holder for this preprint (which was not certified by peer review) is the author/funder, who has granted bioRxiv a license to display the preprint in perpetuity. It is made available under aCC-BY 4.0 International license.

Supplementary Information

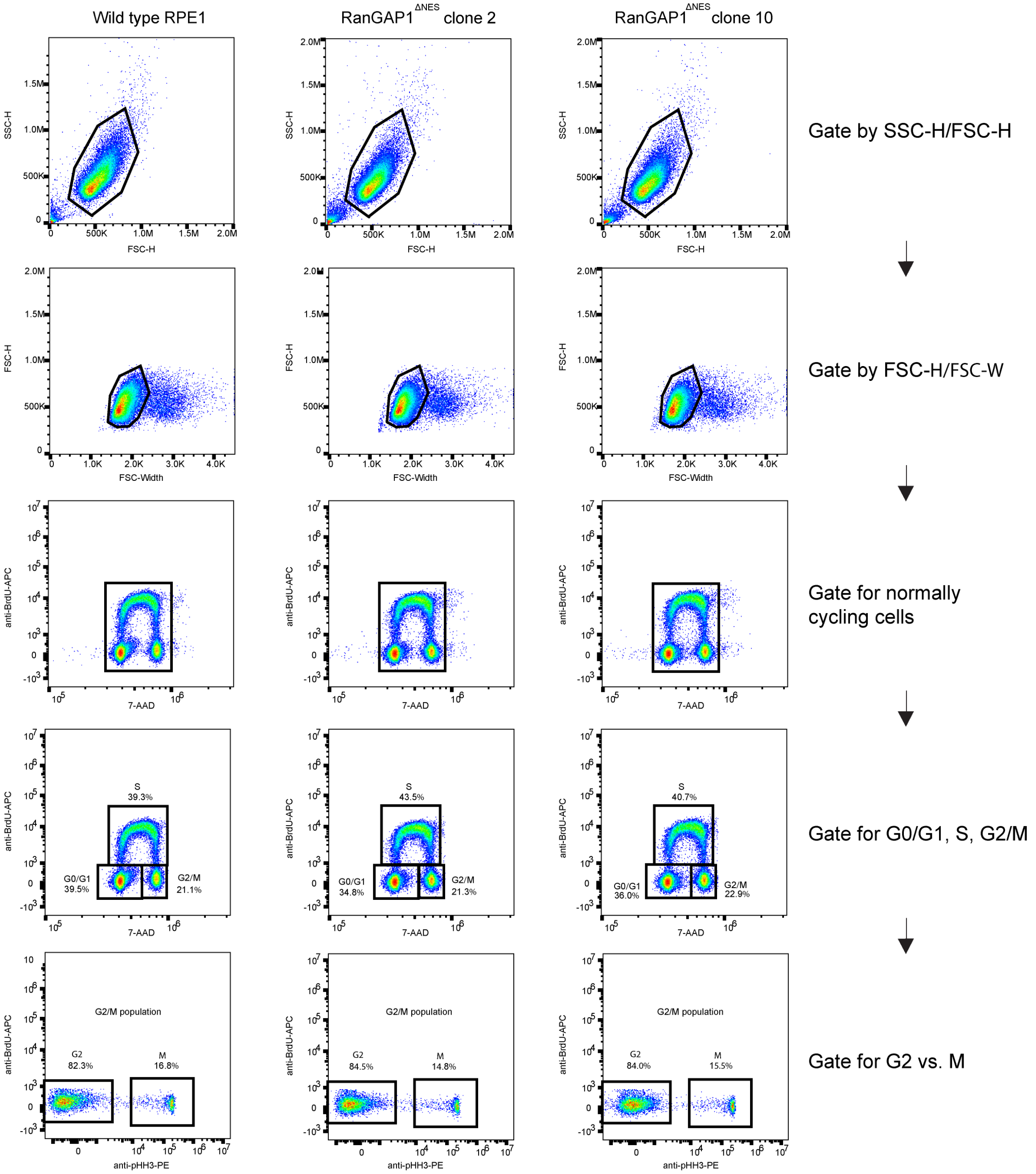

Supplementary Information Figure 1. Representative flow cytometry gating strategy for cell cycle analysis for wild type RPE1 and RanGAP1$^{\Delta NES}$ clones 2 and 10 – related to Extended Data Fig. 6.

bioRxiv preprint doi: https://doi.org/10.1101/2024.10.04.616749; this version posted December 26, 2024. The copyright holder for this preprint (which was not certified by peer review) is the author/funder, who has granted bioRxiv a license to display the preprint in perpetuity. It is made available under aCC-BY 4.0 International license.

Source Data

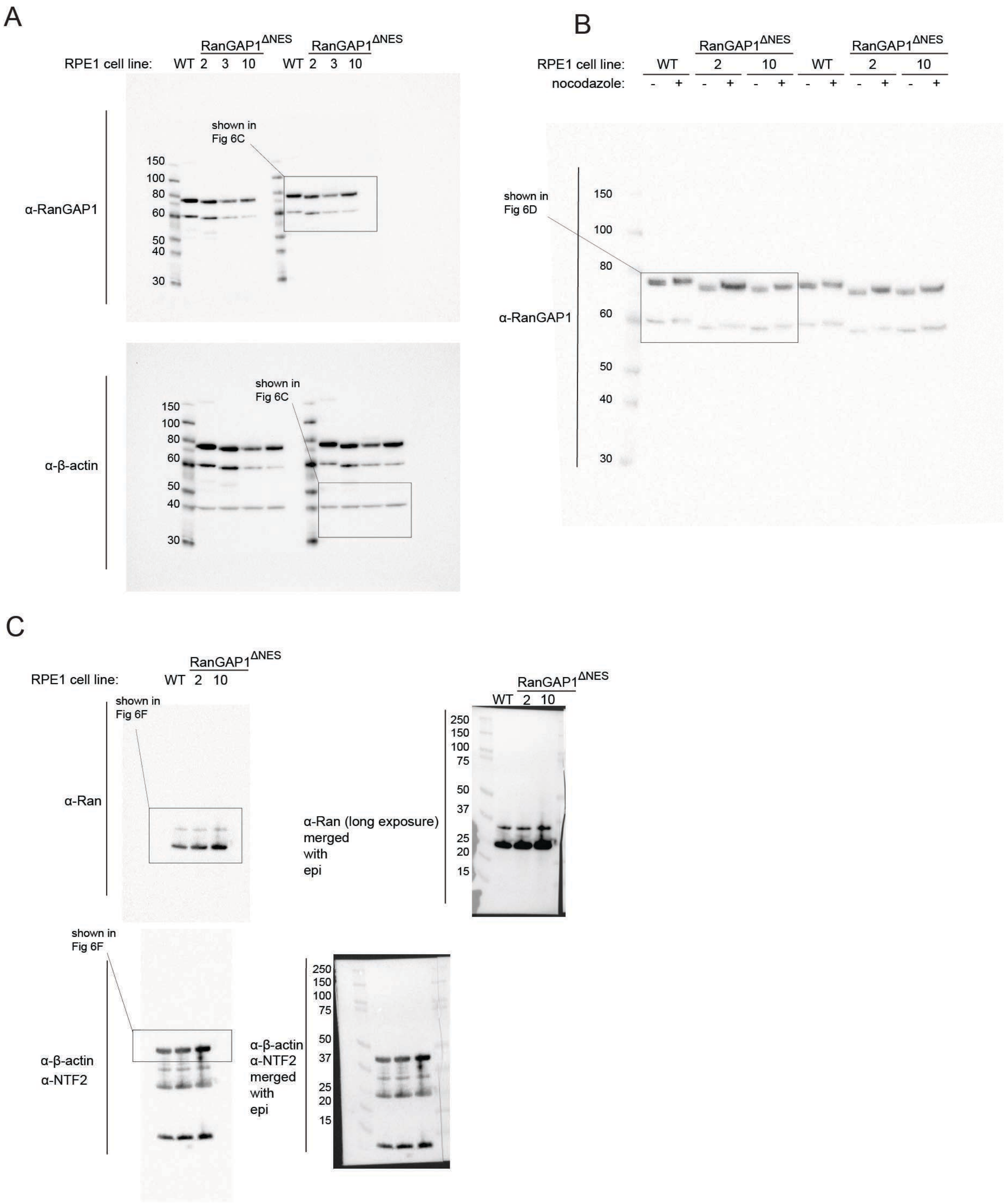

Source Data for Figure 6 - Full Western blots for panels shown in Fig. 6C, D, and F.

bioRxiv preprint doi: https://doi.org/10.1101/2024.10.04.616749; this version posted December 26, 2024. The copyright holder for this preprint (which was not certified by peer review) is the author/funder, who has granted bioRxiv a license to display the preprint in perpetuity. It is made available under aCC-BY 4.0 International license.

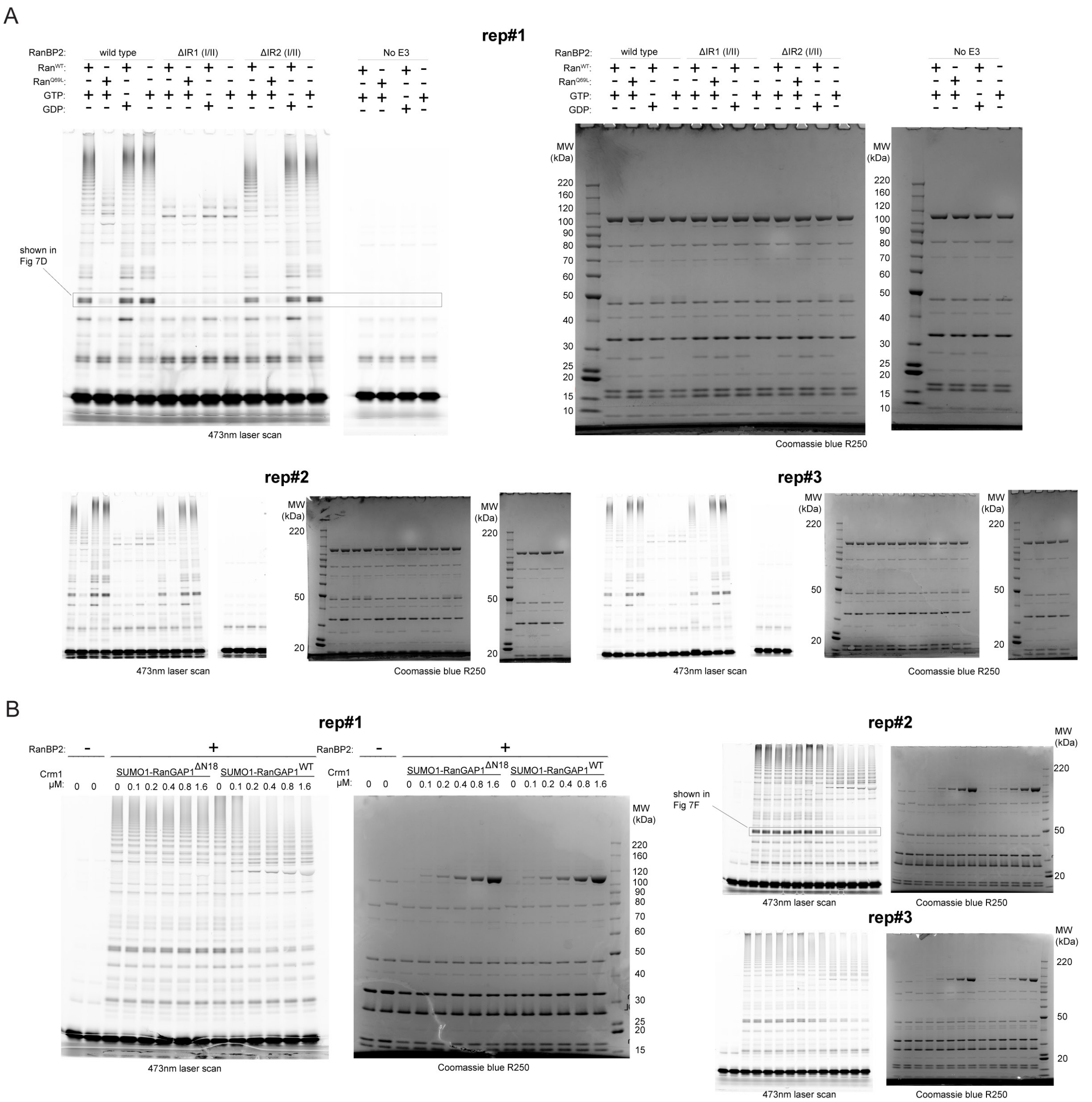

Source Data for Figure 7 - Full gels and associated replicates of data shown in Fig. 7D and F and quantified in 7E and G.
